# Integrating Measures of Fecal Glucocorticoid Metabolites and Giving-Up Densities to Assess Adrenocortical Activity and Well-Being in Zoo-Housed Three-Banded Armadillos (*Tolypeutes matacus*)

**DOI:** 10.3390/ani13121975

**Published:** 2023-06-13

**Authors:** Jennifer Howell-Stephens, Emily J. Potratz, Joel S. Brown, Dave Bernier, Rachel M. Santymire

**Affiliations:** 1 Department of Biological Sciences, University of Illinois at Chicago, Chicago, IL 60607, USArsantymire@gsu.edu (R.M.S.); 2 Department of Integrated Mathematical Oncology, Moffitt Cancer Center, Tampa, FL 33612, USA; 3 Animal Care Department, Lincoln Park Zoo, Chicago, IL 60614, USA; 4 Department of Biology, Georgia State University, Atlanta, GA 30303, USA

**Keywords:** environmental modifications, foraging behavior, human managed care, stress physiology

## Abstract

**Simple Summary:**

To understand how zoo animals perceive their environment, managers evaluate their stress response through behavioral and glucocorticoid analyses. Here, we measured how seven, zoo-housed (Lincoln Park Zoo, Chicago, IL, USA) southern three-banded armadillos (*Tolypeutes matacus*) perceived their habitats using non-invasive sampling of both physiological (via fecal glucocorticoid metabolites, FGMs) and psychological (via foraging behavior) indicators. We offered them depletable food patches with various patch and environmental modifications. Food remaining in a patch represents the animal’s giving-up density (GUD). High GUDs are a response to increased risk; the animal does not feel comfortable and safe to forage. Before, during, and after foraging experiments, feces was collected to examine FGMs. We found that there was no relationship between GUDs and FGMs. However, results varied greatly among individuals, and each individual’s results remained consistent across experiments. Armadillos did not respond to patch modifications but did forage more when the cover substrate was increased. When species do not have dynamic and variable behaviors, as is the case with this species, the evaluation of their perception of the environment can be difficult. For this reason, GUDs and FGMs are practical to offer zoo-housed animals, provide valuable information for zoo managers on husbandry modifications and serve as enrichment for the animal.

**Abstract:**

To monitor adrenocortical activity in zoo-housed species, we propose using physiological and behavioral indicators that are non-invasive and practical to implement. We explore this model in the southern three-banded armadillo (*Tolypeutes matacus*; armadillo), which is a near-threatened species commonly found in zoos. We aimed to (1) deploy food patches to quantify foraging behavior (via giving-up densities, GUDs); (2) determine the effects of food patch and environmental modifications on individuals’ GUDs and adrenocortical activity (via fecal glucocorticoid metabolites, FGMs); and (3) examine the relationship between GUDs and FGMs. Three males and four females received food patches under varying experimental conditions at the Lincoln Park Zoo (Chicago, IL, USA). Fecal samples were collected before, during, and after foraging experiments to examine FGMs. Armadillos did not respond to patch modifications but did forage more when given increased cover. Individual mean FGMs and GUDs were highly variable, and individuals had consistent FGM and GUD ranks across experiments. FGMs and GUDs did not vary across the experiments nor did they relate to each other. Armadillos and species with a limited behavioral repertoire (i.e., constant movement) can benefit from this multi-trait model to determine the effect of environmental modifications on individuals and provide meaningful information about adrenocortical activity.

## 1. Introduction

A major component of assessing zoo animal well-being is considering how animals perceive the zoo environment. While not synonymous, stress physiology and well-being are inextricably linked and can broadly be used to describe how an individual is coping with its conditions [1,2]. To understand stress physiology we can monitor adrenocortical activity. When challenged, an individual’s hypothalamus-pituitary-adrenal (HPA) axis connects the central nervous system with the endocrine system to produce glucocorticoids, a stress-associated hormone, from the adrenal cortex [2,3]. Circulating glucocorticoids play a vital role in metabolic pathways, the immune system, the cardiovascular system, and brain function [3,4,5]. An increase in their concentration after a stressor can represent a robust and fully developed HPA axis. Acute stressors, such as a social introduction or reproductive event, can cause a normal physiological response such as an increase in glucocorticoids that eventually return to baseline [6]. A dysfunctional or chronically activated HPA axis such as being housed with an aggressive conspecific or repeated acute stressors across a lifetime can result in accumulated physiological and somatic damage, producing an elevated allostatic load [7] and ultimately reduced health [6].

In zoos, monitoring adrenocortical activity is especially vital for breeding animals. When glucocorticoids are elevated for prolonged periods of time they can inhibit the release of gonadotropins, which act on the gonads to promote sex hormones and stimulate the production of sperm or ova [8,9,10]. For over 30 years, the adrenocortical activity of zoo animals has been monitored using fecal hormone metabolite analysis [11,12,13]. Although this method requires a proper validation of physiological and biological relevance [12,14], fecal hormone metabolite analysis allows for a non-invasive and longitudinal evaluation of pooled adrenocortical activity [15,16].

A single metric such as adrenocortical activity, however, does not reflect the entirety of an animal’s needs and perceptions [17]. There is a dynamic interplay between physiological mechanisms and adaptive behavioral strategies that can shape a species’ life-history evolution. Just as adrenocortical activity or the animal’s physiological state provides useful indicators of animal well-being, foraging behavior can provide effective and complementary metrics for gaining insights into an animal’s psychological state [18]. Diverse forms of depletable food patches have been successfully used to provide enrichment for zoo-housed animals. Examples include buried food in outdoor enclosures for wombats (*Lasiorhinus latifrons*) [19], food-filled bones for coyotes (*Canis latrans*) [20] and hidden dead mice in enclosures for maned wolves (*Chyrsocyon brachyurus*) [21]. Foraging enrichment is an inexpensive and easy to measure tool that can be used to gather data on how an animal perceives its environment.

In a depletable food patch, food items are hidden or mixed into an inedible substrate, and the animal experiences diminishing returns while foraging from the patch. As the patch depletes, more time or effort is needed to acquire the subsequent food item, requiring the animal to make foraging decisions in real time [22]. An animal will stay and exploit a food patch more thoroughly when they are in a safe or preferred environment than an animal in a risky or less preferred environment. The giving-up density (GUD) is the amount of food remaining in the patch once foraging has stopped. By estimating an animal’s quitting harvest rate, a lower GUD corresponds to lower foraging costs, which can be interpreted as the animal feeling a sense of comfort [22]. Patch use and GUDs have been used to evaluate foraging ecology in several zoo-housed populations [23,24,25], demonstrating that zoo animals have varying expectations of their environment as evidenced by foraging decisions [26] and space use [23]. GUDs are especially useful in species with a limited behavioral repertoire either due to natural ecology or limited space and husbandry in zoo settings.

In this study, we investigated the relationship between adrenocortical activity and foraging behavior to develop a tool for measuring how an animal perceives its environment. The southern three-banded armadillo (*Tolypeutes matacus*; hereafter, armadillo) is a species of interest due to their presence in many zoo environments. The armadillo is a near-threatened insectivore [27] that inhabits the xeric regions of Argentina, Bolivia, Brazil and Paraguay [28]. They have been housed in zoos for over 40 years [29]. A total of 92 institutions accredited by the Association of Zoos and Aquariums (AZA) currently house the species. Unfortunately, the armadillo’s reproductive success is limited due to an offspring mortality rate of 45% (52.8% of males, 37.5% of females) across the AZA institutions [30]. In the wild, such mortality rates are not measurable, and armadillo conservation is primarily threatened from hunting and habitat loss [28]. A reproductive decline in zoo settings can be an indicator of poor well-being, and therefore, having multiple metrics to understanding such states is vital for the armadillo’s health within zoos.

In this study, we aimed to develop a conceptual model for integrating two complementary metrics, adrenocortical activity and foraging behavior, to determine how zoo-housed armadillos perceive their environment. Our objectives were to: (1) develop a food patch that could be used to examine armadillo foraging behavior (via GUDs); (2) determine the effects of food patch modifications (substrate quantity and patch quality) and environmental modification (cover amount) on armadillo GUDs and adrenocortical activity (via fecal glucocorticoid metabolite (FGM) analysis); and (3) examine the relationship between physiological (FGM) and psychological (GUD) states. In studies on ex situ desert gerbils (*Gerbillus andersoni allenbyi* and *Gerbillus nanus*) free-ranging in an outdoor vivarium, GUDs were lower on dark nights (perceived as safer by gerbils) and higher when owls were present [31,32,33]. They found that FGMs and GUDs did not strongly correlate; however, FGMs were also lower on dark nights regardless of whether owls were present or not, but were higher on nights with a full moon when owls were present. Here, when testing how FGMs and GUDs inform on the adrenocortical activity of armadillos, we too predict that both metrics will reveal the animal’s perceived risks and costs. Using both indicators, we can then relate individual armadillos to a state category (high or low GUDs crossed with high or low FGMs) that can gauge how armadillos perceive their zoo environment.

## 2. Materials and Methods

### 2.1. Animals and Housing

Seven (3 males and 4 females) armadillos, housed at Lincoln Park Zoo (LPZ) in Chicago, IL, USA were included in this study. These animals are housed off exhibit in a temperature-controlled holding room exposed to natural and artificial light throughout the year. One armadillo (ID 9717) was used for weekly education programs. The mean (±SE) age of the armadillos was 16.2 ± 3.8 years old (range, 6.5–33.8 years). Animals were housed individually in wooden enclosures that were 112 cm × 58 cm × 61 cm. Enclosures were positioned four in a row so animals shared one to two walls with their neighbor. Armadillos were given ~1.0 kg (i.e., ~2 flakes) of straw dispersed across the whole enclosure for animals to use as cover and to hide underneath.

Armadillos were fed Mazuri insectivore diet**^®^** (PMI Nutrition International, St. Louis, MO, USA) plus chopped vegetables and/or fruits and six wax worm larvae (*Galleria mellonella*) per day. Diets remained consistent across our study, except larvae were excluded from their regular diet as armadillos were given worm larvae as the food item during foraging experiments.

### 2.2. Fecal Sample Collection and Processing

Fecal samples were collected five to seven times per week during the routine cleaning of armadillo enclosures. Sample collection began one month prior to foraging experiments (September 2009), continued during foraging experiments, and ended one week after the completion of all foraging experiments (March 2010; 630 total samples: approx. 90/armadillo). Samples were stored at −20 °C until processing at the LPZ Endocrinology Laboratory. Fecal samples were lyophilized (Labconco Lyophilizer, Kansas City, MO, USA), and hormones were extracted using a previously described protocol [34]. Briefly, dried samples were pulverized, and 0.02 g (±0.002 g) of fecal powder was briefly vortexed with 0.5 mL of 90% ethanol. Samples were shaken (Glas-col mixer, Terre Haute, IN, USA, setting 60, 30 min) and then centrifuged (1500 rpm, 20 min). Extracts were poured off into clean test tubes and the fecal pellets were then re-suspended in 0.5 mL of 90% ethanol and vortexed for 30 s. After centrifugation (1500 rpm, 15 min), extracts were combined with the first extracts and air dried. Samples were then reconstituted in 0.2 mL of phosphate-buffered saline (PBS; 0.01 M PO_4_, 0.14 M NaCl, 0.05% BSA, 0.01% NaN_3_, pH 7), vortexed briefly, sonicated for 20 min and shaken (Glas-col mixer, setting 60, 20 min). Extracted samples were diluted with PBS (1:20) for hormone analysis.

### 2.3. Hormone Analysis

FGMs were analyzed using a cortisol enzyme immunoassay. This assay was previously validated using an adrenocorticotrophic hormone challenge along with biochemical validations of demonstrating both parallelism between binding inhibition curves of fecal extract dilutions (1:2–1:256) and a significant percent recovery (>90%) of exogenous cortisol added to fecal extracts [35]. Cortisol polyclonal antiserum and HRP (R4866; provided by C. Munro, Davis, CA, USA) were used at a 1:8500 and 1:20,000 dilution, respectively [36]. Cross-reactivities to the cortisol antiserum were: cortisol, 100%, prednisolone, 9.9%; prednisone, 6.3%; cortisone, 5%; corticosterone, 0.7%; deoxycorticosterone, 0.3%; 21-deoxycortisone, 0.5%; 11-deoxycortisol, 0.2%; progesterone, 0.2%; 17α-hydroxyprogesterone, 0.2%; pregnenolone, 17α-hydroxypregnenolone, anderostenedione, testosterone, androsterone, dehydroepiandrosterone, dehydroisoandrosterone-3-sulfate, aldosterone, estradiol-17β, estrone, estriol, spironolactone and cholesterol, 0.1% [13]. Assay sensitivity was 3.9 pg/well. Intra- and inter-assay coefficients of variation were <10% and 15%, respectively.

### 2.4. Foraging Patch Procedure and Experiments

**Figure 1:**
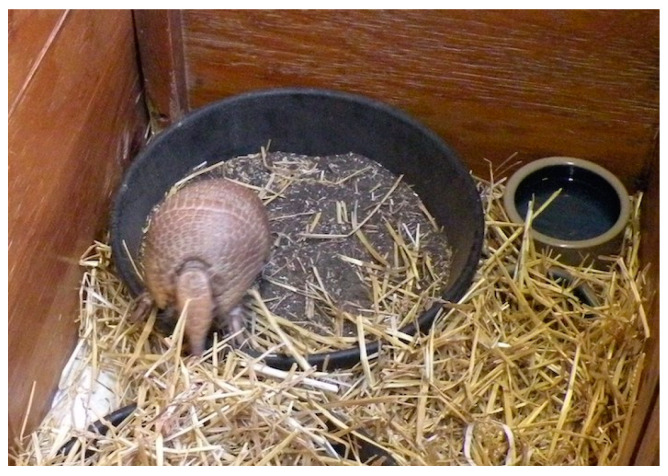
During foraging experiments the armadillos were able to enter the food patch and had straw to use as cover and bedding, as seen here (Photo by JHS).

Foraging experiments were set up in the armadillo’s normal enclosure described above. Patches consisted of 7.6 L black rubber round pans (36.8 cm diameter, 10.2 cm deep), 3.8 L of loose topsoil, and nine larvae mixed randomly into the topsoil (Figure 1). Previously, we had determined larvae were a highly desirable food for the armadillos by placing them into the patch without the topsoil. Each armadillo readily consumed 30 or more worms. Hence, we concluded that any food left behind (GUDs) within food patches with substrate represents a balance between the armadillos’ perceived costs and benefits of foraging. Each armadillo received two patches at the end of the day (1600). We recovered patches the next morning (0800). Any remaining larvae were collected by sieving the soil from the patch and counting the remaining larvae, thus providing the GUD. Prior to the experiments starting, armadillos were given the control patch for four consecutive nights to eliminate any neo-phobic reactions to the foraging patches [37]. First, we tested substrate quantity, then patch quality, and finally cover amount with at least nine days of non-testing time in between in which armadillos did not use food patches nor were fecal samples collected. For each experimental period, all armadillos were measured on the same nights.

#### 2.4.1. Substrate Quantity

To examine if the quantity of substrate within the patch affected foraging, armadillos were given two patches per night, one with 3.8 L and one with 5.7 L of topsoil (each with nine larvae). This experiment was repeated two times a week for two weeks for a total of four replicates.

#### 2.4.2. Patch Quality

To examine if the initial food abundance, or initial prey density (IPD), affected foraging, armadillos were given two patches with the same amount of topsoil (3.8 L) but with either six or 12 larvae. This experiment was repeated two times a week for two weeks for a total of four replicates.

#### 2.4.3. Cover Quantity

To examine if the quantity of cover (straw) affected foraging, we varied the amount armadillos received in their enclosures. One flake was considered low cover, two flakes were considered medium cover, and three flakes were considered a high cover amount (c. 500 g of straw per flake). Armadillos were given two patches with nine larvae and 3.8 L of topsoil for each treatment day. The order each armadillo received the three cover treatments was randomly assigned. Armadillos received each treatment for four consecutive days, resulting in 12 total days of data for each armadillo.

### 2.5. Integrating FGMs and GUDs into States of Stress and Well-Being

A conceptual model was created to integrate FGMs and GUDs into ‘low’ and ‘high’ state categories (Table 1). Low GUDs indicate thorough foraging and low perceived foraging costs, including a sense of security and safety. High GUDs indicate the opposite. Low FGMs represent reduced adrenocortical activity versus high indicating elevated adrenocortical activity. When combined, these metrics can help describe an animal’s state of well-being and ability to cope in the environment. High and low FGM and GUD states can have multiple meanings and explanations. High FGMs can be concerning if no context is available to explain where this rise in adrenocortical activity came from. Is the animal engaging in play or foraging behaviors that may elicit a beneficial physiological stress response? If so, then they are coping in their environment and this would be considered positive. Or does the animal seem uninterested in food or showing maladaptive behaviors? A lack of relationship between FGMs and GUDs supports using both in an orthogonal table to complement each other and provide more context on animal state. One metric is not sufficient.

Determinations of high or low FGM and GUD states were evaluated for each armadillo based on its FGMs and GUDs. Four state categories were created, three of which we believe are acceptable for maintaining and promoting zoo-animal well-being. Low GUDs and low FGMs indicate a near-ideal state. Individuals value foraging thoroughly and exhibit no elevated adrenocortical activity. Low GUDs and high FGMs indicate a positive state where both foraging intensity and adrenocortical activity are elevated as signs of eustress. High GUDs and low FGMs indicate a neutral state for the animal. It reveals limited foraging that is not being driven by environmental stressors. High GUDs and high FGMs indicate a negative state. The individuals are not foraging due to distress from perceived stressors [25].

### 2.6. Data Analysis

#### 2.6.1. Adrenocortical Activity

FGM data were non-normal, and therefore, non-parametric analyses were conducted. First, we ran a Kruskal–Wallis with a Dunn’s method post hoc comparison to compare the overall mean FGMs across individuals. Using R version 4.2.1 [38], statistical analyses and data visualizations were performed with packages FSA [39], agricolae [40] and ggplot2 [41].

To determine if individuals’ FGMs varied across pre-sampling, substrate quantity experiment, patch quality experiment, cover quantity experiment and post-sampling, we used a repeated-measures ANOVA Friedman test of rank sum with the five time periods blocked within individuals. If FGMs were higher (*p* < 0.05) than the previous experiment, then FGM values were considered ‘high’. If experiment FGMs did not differ or were lower (*p* < 0.05) than the previous experiment, then FGMs were considered ‘low’. To determine if FGMs remained consistent within an individual among the three foraging experiments, we assigned individuals’ mean FGM measures for each experiment a rank (range, 1–7), with the lowest FGM mean ranked as 1 and the highest FGM mean ranked as 7, and calculated Kendall’s W coefficient of concordance for rank using Friedman’s test.

#### 2.6.2. Foraging Behavior

We analyzed foraging measures across the three experiments using general linear models. To test for the effect of (1) substrate quantity, (2) patch quality (initial prey density, IPD) and (3) cover quantity on foraging, we used GUDs as the dependent variable, and date, individual, treatment and the interaction between individual and treatment as independent variables in separate ANOVAs for each experiment. Pair-wise comparisons for significant effects were made using a Student–Newman–Keuls test.

For the substrate and patch experiment, single GUDs were reported for each treatment. For the patch quality experiment, we also measured the effect of IPD on proportion harvested. For proportion harvested, GUD measures were transformed to determine the proportion of IPD harvested using 1-GUD/IPD. To test for the effect of IPD on the proportion harvested, we used proportion harvested as the dependent variable, and date, individual, IPD and the interaction between individual and IPD as the independent variables in the ANOVA. To examine the effect of cover quantity, GUDs from both patches for each treatment were summed together to create a total GUD. For all models, the GUDs and total GUDs were square root transformed for residuals to meet assumptions of normality.

To determine if individuals had ‘low’ or ‘high’ GUDs, we compared individual’s GUDs to the mean possible foraging measure. GUDs were considered high if they were greater than half of the highest mean possible GUD measurement (GUD = 4.5, total GUD = 9); otherwise, the GUD measurement was low. To determine if GUDs remained consistent within an individual among the three foraging experiments, we assigned individuals’ mean GUD measures for each experiment a rank (range, 1–7), with the lowest GUD mean ranked as 1 and the highest GUD mean ranked as 7, and calculated Kendall’s W coefficient of concordance for rank using Friedman’s test.

#### 2.6.3. Relationship between FGMs and GUDs

FGMs and GUDs were analyzed using a Spearman rank correlation. GUD measurements had a paired FGM measure that coincided with the fecal sample collected the day after patches were given. This was to account for the time (~24 h) it takes steroids to be metabolized and excreted during gut passage [12]. After the categorization of armadillos into ‘low’ or ‘high’ FGM and GUD values using the methods described above, we determined which of the four states (Table 1) the individual was in and if that state changed across experiments.

## 3. Results

### 3.1. Adrenocortical Activity

Adrenocortical activity results are reported as mean ± SEM FGMs (ng/g of dry feces). Overall, the mean FGMs between individuals varied (H_6_ = 287.73; *p* < 0.01; Table 2). Two of the three males (ID 20202, 6474) had mean FGMs two- and fourteen-fold higher than the other individuals (Table 2). When blocked within individuals, FGMs across all time periods were similar (H_4_ = 4.46; *p* > 0.05; Table 2) for pre-sampling (3160.04 ± 345.23), substrate experiment (3152.63 ± 537.41), patch quality experiment (3403.03 ± 805.81), cover quantity experiment (3260.93 ± 816.95), and post-sampling (2802.59 ± 888.20). The rank ordering of FGMs across individuals remained similar for all three experiments (W = 0.5; *p* > 0.05; Table 3).

### 3.2. Foraging Behavior

**Figure 2:**
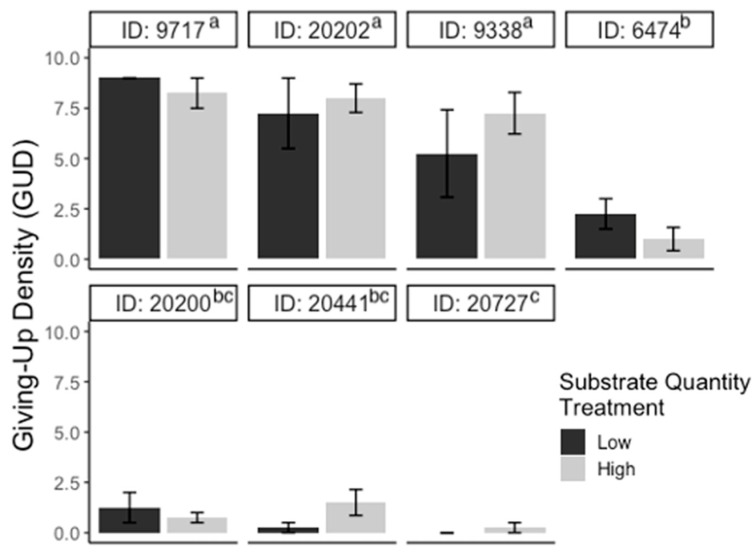
Bar plots of giving-up densities (GUDs) for individuals (ID) during substrate quantity experiment. Vertical lines represent the standard error of the mean. The color of the bar represents the treatment given (black = low substrate, gray = high substrate) and superscripts indicate differences (*p* < 0.05) between individuals.

**Figure 3:**
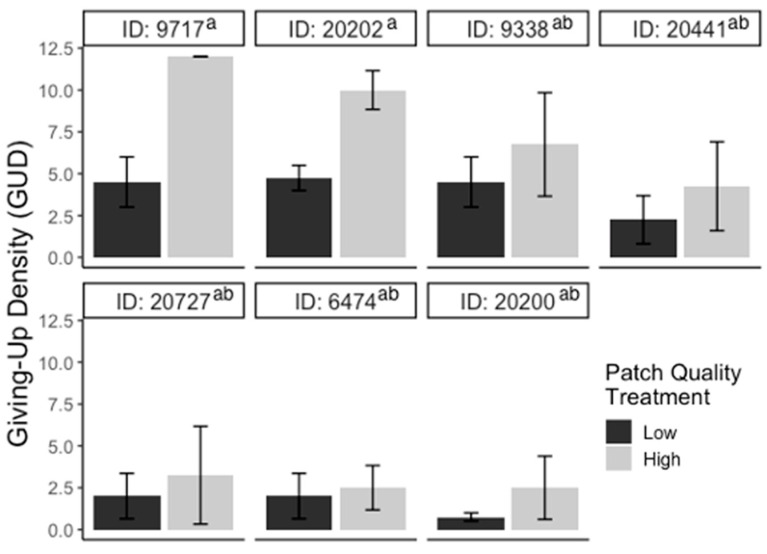
Bar plots of giving-up densities (GUDs) for individuals (ID) during patch quality experiment. Vertical lines represent the standard error of the mean. The color of the bar represents the treatment given (black = low patch, gray = high patch) and superscripts indicate differences (*p* < 0.05) between individuals.

**Figure 4:**
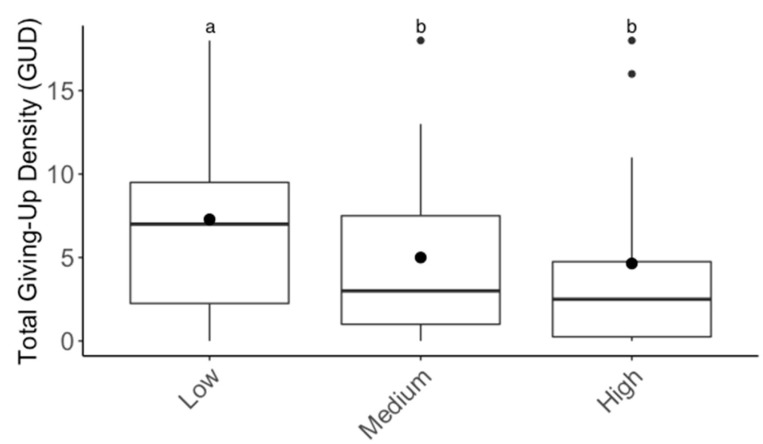
Boxplots of total giving-up densities (GUDs) for each cover treatment (low = 1 flake of straw, medium = two flakes of straw, high = three flakes of straw). Upper whisker = Q3 + (1.5 × IQR) and lower whisker = Q1 *−* (1.5 × IQR). Inside the box, circles denote the mean and the horizontal line denotes the median. Outside the box, circles denote outliers and superscripts indicate differences (*p* < 0.05) between cover treatments.

For the substrate experiment, GUDs varied significantly between individuals (F_6,39_ = 30.10; *p* < 0.001), but there were no significant differences in GUDs between treatments (F_1,39_ = 0.71; *p* > 0.05), dates (F_3,39_ = 2.09; *p* > 0.05), and treatments within individuals (F_6,39_ = 1.45; *p* > 0.05; Figure 2). For the patch experiment, GUDs also only varied by individuals (F_6,39_ = 3.2; *p* < 0.05) and were similar among treatments (F_1,39_ = 3.5; *p* > 0.05), dates (F_3,39_ = 0.14; *p* > 0.05), and treatments within individuals (F_6,39_ = 0.5; *p* > 0.05; Figure 3). Looking at the proportion of IPD harvested, the results are the same: the only variation in foraging is by individual (F_6,39_ = 4.03; *p* < 0.01; Figure S1). For the cover experiment, foraging measures are reported as mean ± SEM total GUD. Total GUDs declined with the increase in cover quantity (F_2,18_ = 6.33; *p* < 0.01, Figure 4). Individuals in low cover treatments had higher total GUDs (7.29 ± 1.48) than individuals given medium (5.00 ± 1.52) and high cover (4.63 ± 1.61), which were similar. Total GUDs also varied by individuals (F_6,18_ = 21.13; *p* < 0.001; Figure S2) and dates (F_5,18_ = 4.23; *p* < 0.05). There was no significant interaction effect between cover amount and individual (F_10,18_ = 1.84; *p* > 0.05).

The rank ordering of GUDs across individuals remained similar for all three experiments (W = 0.4; *p* > 0.05; Table 3). Thus, regardless of experiments, individuals had their own unique and consistent propensity for lower or higher GUDs relative to others.

### 3.3. Relationship between FGMs and GUDs

Regardless of the experiment, FGMs and GUDs did not covary positively or negatively (substrate quantity: r_s_ = −0.06, *p* = 0.8; patch quality: r_s_ = −0.4, *p* = 0.1; cover amount: r_s_ = 0.2, *p* = 0.2). Therefore, we can treat them as somewhat orthogonal and use both metrics to assign individuals to one of the four possible states of well-being and stress. Individuals had their own unique and consistent adrenocortical activity, but there was no detectable increase or decrease in FGMs across time and all animals were considered to have ‘low’ FGMs. Two individuals (ID 20202, 9717) did change from ‘high’ to ‘low’ GUDs during the cover experiment (Table 4) and therefore from ‘neutral’ to ‘ideal’ states. The female (ID 9338) with ‘high’ GUDs throughout the study was in a ‘neutral’ state, while all other animals that had ‘low’ GUDs throughout the study were in an ‘ideal’ state (Table 4).

## 4. Discussion

This study investigates the relationship between adrenocortical activity and foraging behavior of zoo-housed armadillos to create a framework for addressing ex situ animal stress responses and well-being. Ex situ environments try to provide safe and climatically favorable conditions. Yet, zoo-housed animals may still behave in ways shaped by evolution within their native habitats [42]. For example, costs of predation are negligible in zoos, although the animals may still perceive a high predation risk due to the sensory (i.e., visual, olfactory, auditory) stimuli of a predator species or innate fear of novel environments. The armadillos in this study were housed in a room adjacent to a sand cat (*Felis margarita*) enclosure. While we did not have the opportunity to experiment with how sensory cues from a nearby predator influenced the animals, this should be considered as it can provide context to the variation in FGMs and GUDs: if the sand cats were removed, lowered FGMs and lowered GUDs could be indicators of these perceived dangers diminishing. Therefore, zoological institutions could benefit from using multi-trait methodologies for evaluating how an animal is coping within its environment.

We successfully deployed a depletable food patch to gain direct insight into the armadillo’s perceptions of its environment. Patch modifications such as the amount of substrate within a patch were expected to decrease the quantity of food items harvested [43,44] and increase GUDs. However, armadillos did not bias their foraging toward shallower patches, but they had similar GUDs in both patches despite the substrate amount. There are three broad categories of patch use behaviors. Using a fixed amount strategy, the expectation would be to observe the proportion of food harvested declining with initial abundance. A fixed time patch use strategy predicts no effect of initial food abundance on the proportion of food harvested [45]. A fixed quitting harvest rate strategy should result in the proportion harvested increasing with initial abundance. Additionally, the initial abundance of food did not influence the proportion of food harvested or the GUD in the patch quality experiment. Despite their good senses of smell and sight [46], armadillos seemed to be using a fixed time foraging strategy that is most frequently observed in low variance environments [45]. This could be the most optimal behavior for foragers that cannot assess patch quality and characteristics [47], although it means over-utilizing poor patches [45]. Individuals, however, varied significantly in the proportion of food harvested and their overall GUDs. In a lab foraging experiment, wild caught screaming armadillos (*Chaetophractus vellerosus*) took more food items from high compared to low IPD patches [48]. These patches were small containers containing food but no substrate and therefore did not pose a foraging challenge for the armadillos. It could be possible that if these experiments, in our study, had run for more days, the armadillos may have learned to detect patch depth or quality and begun to shift toward a quitting harvest rate patch-use strategy [44].

Increasing the amount of straw for cover and bedding resulted in an increase in foraging and lower GUDs. Most likely, more cover provides animals such as these armadillos with safety from potential predators. Wild armadillos can be found foraging in the open but use burrows for shelter and protection from predators [49]. In this captive setting, increased cover may make the armadillos feel less exposed, decrease their perception of risk, and increase their well-being. This study included one armadillo (ID #9717) that was periodically used for education programs at the zoo and was one of two armadillos to change states from having high GUDs to low GUDs during the cover experiment. Regardless of changes or lack thereof in FGM states, low GUDs can indicate that the animal is in an ‘ideal’ or a ‘positive’ state, feeling more comfortable and foraging more thoroughly. Providing ample cover, especially for education program animals, can address states of stress and well-being, and it has been implemented in current husbandry practices at the zoo.

GUDs and FGMs varied across individuals, but an individual’s overall ranking remained consistent across the experiments. This supports the opportunity for combining assessments to better understand individual-specific responses to their environment. Choosing individuals that display specific behaviors (active vs. passive) may relate to their adrenocortical and foraging. Previous work examining the armadillo’s adrenocortical activity demonstrated that FGMs varied among individuals, but they were similar across demographic factors (e.g., sex, age and birth location) [35]. Individual differences in adrenocortical activity may be attributed to differing personality types in the armadillos. These differences should be further investigated, as individual armadillos may react differently to environmental features and to stressors. Welfare may be improved by customizing husbandry and management to the individual [50,51,52,53], which is often based on demographic factors.

We found no relationship between individual’s GUDs and FGMs. Thus, both metrics may serve as complementary indicators of stress and well-being that can be used by zoo managers to gauge husbandry needs. While we did not test for physiological changes via adrenocortical activity within the experiments, another study of zoo-housed armadillos used in education programs found individual FGMs decreased when the depth of enclosure substrate increased [54]. Taken together with our results, increasing foraging (low GUDs) and decreasing adrenocortical activity (low FGMs) can result in an ideal state of stress and well-being that zoo managers may aim for. In this ideal state, the animal perceives much to be gained from foraging, perceives little risk, and has acclimated well to its exhibit space. Individuals with high GUDs and low FGMs may be in a neutral state. Such an animal may perceive little benefit from foraging yet low perceived stressors. For example, elevated ambient temperatures could reduce foraging as a result of high metabolic costs, but the individual is not physiologically stressed. Zoo-housed American bison (*Bison bison bison*) and Grant’s zebras (*Equus burchelli*) exhibited high GUDs on days with extreme temperatures, showing that they perceived the cost of body temperature regulation to be greater than the benefit that could have been gained from foraging [23]. High GUDs and high FGMs may be a negative state indicating a fearful animal in distress. A high perceived predation cost may result from the exhibit itself or be due to olfactory or auditory cues received from other zoo-housed animals. Animals may perceive higher risks (high GUDs) near exhibit borders (rock hyrax, *Procavia capensis*, and bison) or close to blocked sight lines (zebra) [23]. Low GUDs and high FGMs may represent a positive state and reveal an individual experiencing a normal stress response due to the sensory experience of foraging in a more natural context or an individual coping with a physiological challenge.

Conducting these foraging experiments provides a form of behavioral enrichment, which is an important tool already used to increase the well-being of zoo-housed animals [55]. Providing species-appropriate behavioral opportunities based on an animal’s natural and individual history is critical to obtaining optimal physiological and psychological well-being in zoo-housed species [56]. Foraging accounts for the majority of activity budgets in many wild animals, and it is important to provide zoo-housed animals with opportunities to ‘work’ for their food (i.e., providing bamboo shoots for zoo-housed pandas (*Ailuropoda melanoleuca*) [57]). Feeding enrichment generally evokes a sensory experience by encouraging natural foraging behaviors including an increase in foraging times. Changing a small portion of African elephants (*Loxodonta africana*) diet to a food requiring greater handling time significantly decreased time spent inactive [58]. In large felids, providing live fish and large bones significantly increased the variety of feeding behaviors [59]. Customized food patches can be used to induce natural foraging behaviors [60] and gain insight into the animal’s well-being. Here, foraging patches provided armadillos with the chance to dig and root in soil for insect larvae, allowing them to interact and work for their food in a natural way [61].

## 5. Conclusions

We conclude that the zoo-housed armadillo could benefit from continuing the use of the food patches and fecal hormonal metabolite monitoring to determine how environmental factors affect an individual’s stress and well-being. Zoo institutions could use this tool to evaluate and prioritize planned exhibit and management changes. For example, monitoring GUDs and FGMs can assess the effect of enclosure size on stress physiology and well-being not just in the armadillo but for other species of concern. GUDs can be especially informative in species with limited behavioral diversity, such as constant pacing and movement. Foraging behavior reveals the effort they are willing to put into acquiring the food and hence their perceptions of the costs and benefits posed by their zoo circumstances. Comparing the results across multiple institutions could also provide a unique opportunity to assess the range of individual differences within a species, allow for direct management comparisons and facilitate the sharing of knowledge regarding husbandry improvements.

## Figures and Tables

**Table 1:** The conceptual model framework for combining adrenocortical activity (measured via fecal glucocorticoid metabolites, FGMs) and foraging behaviors (giving-up densities, GUDs), which are bolded, into an overall assessment of an armadillo’s stress and well-being state, which is italicized.

	Low FGM	High FGM
**Low GUD**	*Ideal* Low foraging costs and risk; low adrenocortical activity	*Positive* Low foraging costs; high adrenocortical activity (i.e., eustress)
**High GUD**	*Neutral* High foraging costs; low adrenocortical activity	*Negative* High foraging costs; high adrenocortical activity (i.e., distress)

**Table 2:** Mean ± SEM fecal glucocorticoid metabolites (ng/g dry feces; FGM) of sampling periods by individuals, separated by sex. Samples were collected pre-experiments, during the substrate, patch, and cover experiments and post-experiments. Superscripts represent differences (*p* < 0.05) among individuals.

	ID	Pre	Substrate	Patch	Cover	Post	Overall
**Males**	6474 ^a^	13,130.51 ± 1100.32	12,709.31 ± 1434.52	18,196.97 ± 4084.78	14,757.69 ± 3632.16	17,442.22 ± 2644.83	14,145.06 ± 946.17
	20200 ^d^	742.37 ± 46.65	1004.24 ± 110.44	904.85 ± 97.02	843.49 ± 137.89	1020.97 ± 188.67	848.19 ± 41.27
	20202 ^b^	2504.59 ± 324.92	2646.27 ± 439.17	3451.29 ± 329.20	1935.20 ± 301.07	1412.37 ± 128.09	2457.38 ± 189.83
**Females**	9338 ^c^	1161.40 ± 80.39	1231.13 ± 110.66	994.27 ± 79.75	1073.29 ± 167.89	1061.03 ± 184.70	1132.60 ± 51.21
	9717 ^c^	1112.98 ± 283.66	1023.73 ± 160.44	1294.27 ± 189.11	864.11 ± 124.55	1006.01 ± 181.97	1119.47 ± 143.70
	20441 ^c^	995.49 ± 77.20	1903.70 ± 556.53	1080.93 ± 173.97	1186.18 ± 181.64	963.39 ± 134.78	1167.09 ± 102.28
	20727 ^d^	836.25 ± 38.82	788.19 ± 45.86	889.48 ± 81.73	884.01 ± 97.42	636.78 ± 87.26	830.79 ± 27.91
	Overall	3160.04 ± 345.23	3152.63 ± 537.41	3403.04 ± 805.81	3260.93 ± 816.95	2802.59 ± 888.20	–

**Table 3:** Rank (range, 1–7) order of all individuals for adrenocortical and foraging measures. Individuals with the lowest fecal glucocorticoid metabolites (FGM) or foraging measures (GUD) ranked 1 and individuals with highest measures ranked 7.

		Substrate		Patch		Cover	
	ID	FGM	GUD	FGM	GUD	FGM	GUD
**Males**	6474	7	4	7	2	7	5
	20200	2	3	2	1	1	4
	20202	6	6	6	6	6	6
**Females**	9338	4	5	3	5	4	7
	9717	3	7	5	7	2	3
	20441	5	2	4	4	5	2
	20727	1	1	1	3	3	1

**Table 4:** High vs. low categories of giving-up densities (GUDs) for each armadillo (ID) across the experiments. Asterisk denotes individuals that changed states across experiments.

	ID	Substrate	Patch	Cover
**Males**	6474	Low	Low	Low
	20200	Low	Low	Low
	20202 *	High	High	Low
**Females**	9338	High	High	High
	9717 *	High	High	Low
	20441	Low	Low	Low
	20727	Low	Low	Low

## Data Availability

These data are available upon request made to the corresponding author, E. Potratz.

## Supplementary Materials

The following supporting information can be downloaded at: https://www.mdpi.com/article/10.3390/ani13121975/s1, Figure S1: Bar plots of mean proportion of initial prey density (IPD) harvested for individuals (ID) during patch quality experiment. Vertical lines represent the standard error of the mean. The color of the bars represents the treatment given (black = low patch, gray = high patch) and superscripts indicate differences (*p* < 0.05) between individuals; Figure S2: Boxplots of total giving-up densities (GUDs) of individuals during the cover experiment. Upper whisker = Q3 + (1.5 × IQR) and lower whisker = Q1 − (1.5 × IQR). Inside the box, circles denote the mean and the horizontal line denotes the median. Outside the box, circles denote outliers and superscripts indicate differences (*p* < 0.05) between individuals.
