# Supplementary material for: Integrating Measures of Fecal Glucocorticoid Metabolites and Giving-Up Densities to Assess Adrenocortical Activity and Well-Being in Zoo-Housed Three-Banded Armadillos (Tolypeutes matacus)
Source: Animals (Basel). 2023 Jun 13;13(12):1975. doi: 10.3390/ani13121975 (PMC10295497; doi:10.3390/ani13121975)
Supplement: Supplementary file 1 [file animals-13-01975-s001.zip › animals-2408029-supplementary.pdf]

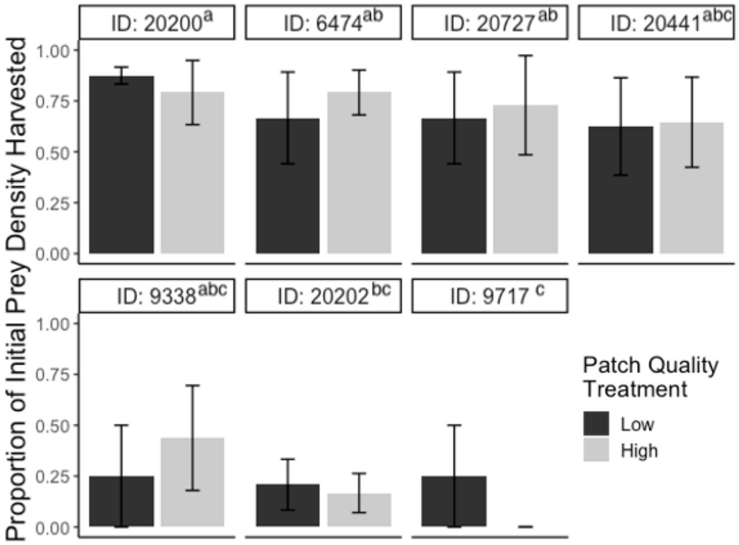

Supplementary Figure S1. Bar plots of mean proportion of initial prey density (IPD) harvested for individuals (ID) during patch quality experiment. Vertical lines represent the standard error of the mean. The color of the bar represents the treatment given (black = low patch, gray = high patch) and superscripts indicate differences ($p < .05$) between individuals.

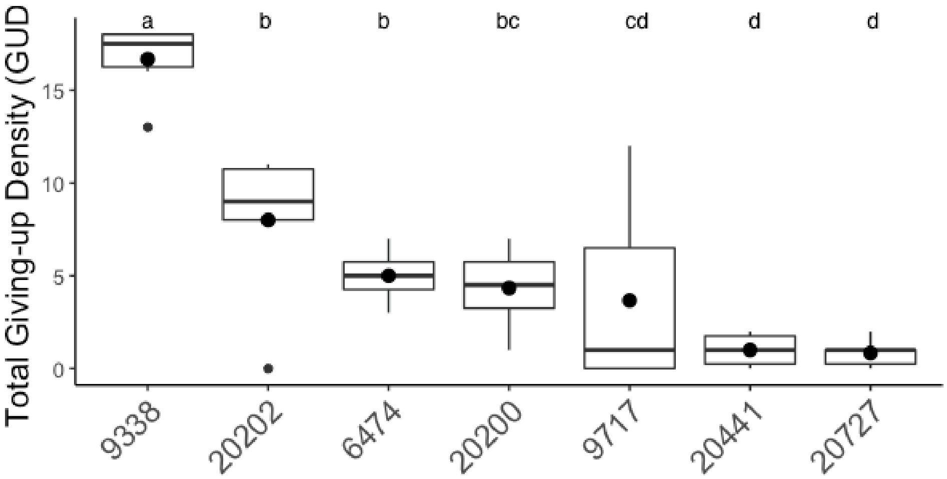

Supplementary Figure S2. Boxplots of total giving-up densities (GUDs) of individuals during cover experiment. Upper whisker = Q3 + (1.5 × IQR) and lower whisker = Q1-(1.5 × IQR). Inside the box, circles denote the mean and the horizontal line denotes the median. Outside the box, circles denote outliers and superscripts indicate differences ($p < .05$) between individuals.
